# Supplementary material for: Impact of the Mbarara University of Science and Technology residency training on increasing access to specialty care workforce
Source: PLOS Glob Public Health. 2024 Mar 20;4(3):e0003021. doi: 10.1371/journal.pgph.0003021 (PMC10954149; doi:10.1371/journal.pgph.0003021)
Supplement: Attachment — Submitted filename: RESPONSE TO REVIEWERS.docx [file pgph.0003021.s003.docx]

**RESPONSE TO ACADEMIC EDITOR’S COMMENTS**

**Journal Requirements:**

1. **Please review your reference list to ensure that it is complete and correct. If you have cited papers that have been retracted, please include the rationale for doing so in the manuscript text, or remove these references and replace them with relevant current references. Any changes to the reference list should be mentioned in the rebuttal letter that accompanies your revised manuscript. If you need to cite a retracted article, indicate the article’s retracted status in the References list and also include a citation and full reference for the retraction notice.**

Response: We have reviewed our reference list, it is complete and correct. We have not cited retracted papers in our manuscript. We have however, added references ((Meyer et al., 2022) and (Cunningham et al., 2015)) regarding to the reviewer’s comment about the response rates in the limitations section of the manuscript on Page 16. This was in response to the question from the reviewer, **“How do your response rates compare to other provider surveys?”** “Our survey response rate was low, at 34.3%, compared to surveys conducted among doctors in general (averaging at 53%) (Meyer et al., 2022), but comparable to specialist response rates to web-based surveys (35.0%) citing the lack of time to respond to the surveys (Cunningham et al., 2015).”

2. **Please include a complete copy of PLOS’ questionnaire on inclusivity in global research in your revised manuscript. Our policy for research in this area aims to improve transparency in the reporting of research performed outside of researchers’ own country or community. The policy applies to researchers who have travelled to a different country to conduct research, research with Indigenous populations or their lands, and research on cultural artefacts. The questionnaire can also be requested at the journal’s discretion for any other submissions, even if these conditions are not met.  Please find more information on the policy and a link to download a blank copy of the questionnaire here: https://journals.plos.org/globalpublichealth/s/best-practices-in-research-reporting. Please upload a completed version of your questionnaire as Supporting Information when you resubmit your manuscript.**

Response: The PLOS’ questionnaire on inclusivity in global research has been completed and is attached. As much as our research involved respondents currently working in diverse environments including other countries and was conducted online, we did not travel to a different country to conduct research. We also assumed that the participants were alumni of a University in Uganda.

3. **Please provide separate figure files in .tif or .eps format only and remove any figures embedded in your manuscript file. Please also ensure all files are under our size limit of 10MB. For more information about figure files please see our guidelines: https://journals.plos.org/globalpublichealth/s/figures**
   [**https://journals.plos.org/globalpublichealth/s/figures#loc-file-requirement**](https://journals.plos.org/globalpublichealth/s/figures#loc-file-requirement)

Response: We have provided separate figure files in .tif format. The respective figures previously embedded in the manuscript have been removed as guided. All files are under the limit of 10MB

4. **We do not publish any copyright or trademark symbols that usually accompany proprietary names, eg  ©, ®, ™  (e.g. next to drug or reagent names). Please remove all instances of trademark/copyright symbols throughout the text, including ® on page 19.**

Response: Thank you for your careful review of our manuscript. We have removed ® on page 19 as suggested.

5. **Some material included in your submission may be copyrighted. According to PLOS’s copyright policy, authors who use figures or other material (e.g., graphics, clipart, maps) from another author or copyright holder must demonstrate or obtain permission to publish this material under the Creative Commons Attribution 4.0 International (CC BY 4.0) License used by PLOS journals. Please closely review the details of PLOS’s copyright requirements here: PLOS Licenses and Copyright. If you need to request permissions from a copyright holder, you may use PLOS's Copyright Content Permission form.**

**Please respond directly to this email or email the journal office and provide any known details concerning your material's license terms and permissions required for reuse, even if you have not yet obtained copyright permissions or are unsure of your material's copyright compatibility.**

**Potential Copyright Issues: Figs 2 & 3: please (a) provide a direct link to the base layer of the map (i.e., the country or region border shape) and ensure this is also included in the figure legend; and (b) provide a link to the terms of use / license information for the base layer image or shapefile. We cannot publish proprietary or copyrighted maps (e.g. Google Maps, Mapquest) and the terms of use for your map base layer must be compatible with our CC-BY 4.0 license.**

**Note: if you created the map in a software program like R or ArcGIS, please locate and indicate the source of the basemap shape file onto which data has been plotted. If your map was obtained from a copyrighted source please amend the figure so that the base map used is from an openly available source. Alternatively, please provide explicit written permission from the copyright holder granting you the right to publish the material under our CC-BY 4.0 license. Please note that the following CC BY licenses are compatible with PLOS license: CC BY 4.0, CC BY 2.0 and CC BY 3.0, meanwhile such licenses as CC BY-ND 3.0 and others are not compatible due to additional restrictions. If you are unsure whether you can use a map or not, please do reach out and we will be able to help you. The following websites are good examples of where you can source open access or public domain maps: 
* U.S. Geological Survey (USGS) - All maps are in the public domain. (http://www.usgs.gov) 
* PlaniGlobe - All maps are published under a Creative Commons license so please cite “PlaniGlobe, http://www.planiglobe.com, CC BY 2.0” in the image credit after the caption. (http://www.planiglobe.com/?lang=enl) 
* Natural Earth - All maps are public domain. (http://www.naturalearthdata.com/about/terms-of-use/)
"**

Response: Thank you for the guidance. Figs 2 & 3: we used OpenStreetMap shape files from Geofabrik an openly available source. Accessible on their data server at [http://download.geofabrik.de](http://download.geofabrik.de/). Specifically we used the base map of Africa accessible on the link: <http://download.geofabrik.de/africa.html>. The direct link to the base layer of the map used has also been included in the figure legend.

The terms of use/license information for the base layer image or shape file are accessible on the link: [http://download.geofabrik.de](http://download.geofabrik.de/), “Data/Maps Copyright 2018 [Geofabrik GmbH](http://www.geofabrik.de/) and [OpenStreetMap Contributors](http://www.openstreetmap.org/) | [Map tiles: Creative Commons BY-SA 2.0](http://creativecommons.org/licenses/by-sa/2.0/) [Data: ODbL 1.0](http://opendatacommons.org/licenses/odbl/) | [Contact](http://www.geofabrik.de/geofabrik/contact.html) | [Legal Notice/Privacy Policy](http://www.geofabrik.de/geofabrik/imprint.html)”. As per the guidance above this fits the CC BY licenses compatible with PLOS license.

**Additional Editor Comments (if provided):**

**Please make changes as suggested by the reviewer to emphasize that this is a convenience sample.**

Response: This is noted. We have emphasized the convenience sample in the abstract and the methods section of the manuscript as suggested by the reviewer below.

**RESPONSE TO REVIEWERS’ COMMENTS**

**General**

1. **This is a clear, well-written manuscript. Its main contribution is in describing a convenience sample of providers that were trained in a residency program. I think the authors could emphasize they have a convenience sample a bit more clearly and earlier in the manuscript, because this could explain the positive affect of the responses. Regardless of this limitation, I think it makes some nice points about HRH satisfaction, geographic distribution of providers following graduation and the reasons for satisfaction or not.**

Response: Thank you for your careful review of our manuscript. We have emphasized the convenience sample in the abstract and the methods section of the manuscript.

**Specific**

2. **Abstract: you should include the number of qualitative interviews here too. I would describe your sample as a convenience sample in the abstract as well.**

Response: We have included the number of qualitative interviews in the abstract and stated that we enrolled a convenience sample of all clinical residency alumni from Mbarara University of Science and Technology (MUST).

3. **How do your response rates compare to other provider surveys?**

Response: Our survey response rate was low, at 34.3%, compared to surveys conducted among doctors in general (averaging at 53%) (Meyer et al., 2022), but comparable to specialist response rates to web-based surveys (35.0%) citing the lack of time to respond to the surveys (Cunningham et al., 2015). However low, our survey response rate would be considered appropriate to provide confident estimates (Wu et al., 2022, Deutskens et al., 2004). This has been updated in the limitations section of the manuscript.

4. **It is likely that you have (self)-selection bias in both the online survey participation as well as the in-depth interview participation and I would postulate it biases the results toward those more satisfied with the program. How might you address this selection bias or do so in future studies? For example, phone follow-up would be a way to increase response rates. The low response rates will be important to mention this as a limitation of the study.**

Response: Thank you for your careful review of our manuscript. We noted this bias in our limitations, “The survey was voluntary and anonymous, this may be prone to sampling bias—respondents with a smooth transition to employment and in a good professional situation may be more willing to take the survey than those who had difficult transition to employment and/or poor professional situation”.

We could address the selection bias in future studies, by stratifying by the different specialties, providing incentives for response to the survey, and phone follow-ups to improve the response rates as suggested.

5. **Is there anything about the program that emphasizes OB/GYN? Or is this a feature of the job market? Providers’ interests?**

Response: The OB/GYN program at MUST has the biggest number of the trainees accounting for 30% of all the residents. I would imagine it’s partly a feature of providers’ interests.

6. **Are there differences in job satisfaction between providers in the public vs. private sectors?**

Response: Overall, there was no significant difference in satisfaction among those in public versus private, a bigger proportion were satisfied (37% [public] versus 49% [private]) or very satisfied (30% [public] versus 29% [private sector]), (p<0.460). This was not different for the other assessed parameters. This information has been in the results section.

7. **Figure 6: you should complete this label: “Possibilities to use qualifications acquired during my…”**

Response: Thank you for your careful review of our manuscript. We have edited the label from “Possibilities to use qualifications acquired during my studies” to “Possibilities to use the acquired qualifications” so that fits in the label space.

8. **Quotations: There are a few typos you should address.**

Response: The typos have been addressed in the quotations
